# Conservation Genetics of an Endangered Lady’s Slipper Orchid: *Cypripedium japonicum* in China

**DOI:** 10.3390/ijms150711578

**Published:** 2014-06-30

**Authors:** Xin Qian, Quan-Jian Li, Fen Liu, Mao-Jiang Gong, Cai-Xia Wang, Min Tian

**Affiliations:** 1 Research Institution of Subtropical Forestry, Chinese Academy of Forestry, Fuyang 311400, China; E-Mails: qianxin090@163.com (X.Q.); gmj0718joe@163.com (M.-J.G.); cxwanghn@163.com (C.-X.W.); 2 Shanghai Chenshan Plant Sciences Research Center, Chinese Academy of Sciences, Shanghai Chenshan Botanical Garden, Shanghai 201602, China; E-Mail: lqj170209@163.com; 3 South China Botanical Garden, Chinese Academy of Sciences, Guangzhou 510650, China; E-Mail: liufen0424@126.com

**Keywords:** *Cypripedium japonicum*, genetic diversity, ISSR, *Orchidaceae*

## Abstract

Knowledge about the population genetic variation of the endangered orchid, *Cypripedium japonicum*, is conducive to the development of conservation strategies. Here, we examined the levels and partitioning of inter-simple sequence repeat (ISSR) diversity (109 loci) in five populations of this orchid to gain insight into its genetic variation and population structure in Eastern and Central China. It harbored considerably lower levels of genetic diversity both at the population (percentage of polymorphic loci (*PPL*) = 11.19%, *Nei’s gene diversity* (*H*) = 0.0416 and *Shannon’s information index* (*I*) = 0.0613) and species level (*PPL* = 38.53%, *H* = 0.1273 and *I* = 0.1928) and a significantly higher degree of differentiation among populations (*the proportion of the total variance among populations* (*Φ_pt_*) = 0.698) than those typical of ISSR-based studies in other orchid species. Furthermore, the Nei’s genetic distances between populations were independent of the corresponding geographical distances. Two main clusters are shown in an arithmetic average (UPGMA) dendrogram, which is in agreement with the results of principal coordinate analysis (PCoA) analysis and the STRUCTURE program. In addition, individuals within a population were more similar to each other than to those in other populations. Based on the genetic data and our field survey, the development of conservation management for this threatened orchid should include habitat protection, artificial gene flow and *ex situ* measures.

## 1. Introduction

Population genetic analyses in the field of biological conservation have been the topic of considerable discussion; they are increasingly emphasized, because assessments of different levels of genetic diversity and population structure within species can increase the understanding of their evolutionary history and population dynamics, and they are also critical for developing effective conservation management strategies [1]. Orchids are a flagship group in plant conservation [2]. The Chinese orchid flora is distinguished by its rich diversity in geographical types, and it includes about 1247 species and 171 genera [2]. The existing evidence demonstrates that most Chinese orchid species have suffered dramatic declines in abundance, mostly due to over-collection and increased habitat fragmentation and destruction [3]. This reduces population sizes, which may then lead to reduced gene flow and increased levels of inbreeding through random genetic drift, with a consequent loss of genetic diversity [4]. The long-term survival of any species requires the maintenance of sufficient genetic diversity to serve as the raw material for evolutionary changes in natural populations [5]. Brzosko *et al*. [6] suggested that small and fragmented populations usually have lower genetic diversity and are more vulnerable to demographic, environmental and genetic stochasticity, which raises their risk of extinction. Therefore, it is very important to understand the level of genetic variation and the degree of divergence among populations and, in particular, the extent to which those differences will affect survival and reproduction in various habitats [7]. From a conservation point of view, knowledge about a species’ genetic diversity has a predictive value for the development of appropriate and effective conservation plans [8]. Swarts *et al*. [9] pointed out that conservation genetics provides theoretical guidance and practical applications to preserve diversity in natural populations of rare and threatened orchids.

*Cypripedium japonicum* Thunb. is a 35–55 cm-tall lady’s slipper orchid that grows in damp and humus-rich soil in forests or on shady slopes along ravines at an altitude of 1000–2000 m; it is mainly distributed in China, Japan and Korea [10]. It is also a diploid, self-compatible, non-rewarding species that bears a solitary yellowish-green flower and has long, creeping rhizomes with rather stout roots formed at the nodes; so, it reproduces both sexually and vegetatively [11]. In China, *C. japonicum* is at great risk of extinction due to habitat loss and anthropogenic threats, such as over-collection for its high horticultural potential and limited medicinal value. Consequently, this species is categorized as a conservation priority plant species [12]. To date, some efforts have been made to elucidate its pollination biology [13] and reproductive characteristics [11]. However, there are still few reports of the population genetics of this endangered species. Chung *et al*. [14] found a complete lack of allozyme diversity between six *C. japonicum* populations in South Korea, and there is only one report describing the use of DNA molecular markers to reveal the genetic diversity of wild *C. japonicum* populations in Zhejiang province [15]. The genetic diversity and structure of this species in other parts of China have not been widely investigated.

The maintenance or restoration of genetic diversity is now regarded as a primary objective for conserving endangered and threatened species, which has resulted in the application of molecular methods and phylogenetic studies to design and implement conservation strategies [9]. Because of no affect by environmental or biological factors, the use of molecular markers to characterize genetic diversity and structure has become an important and effective tool to study population genetics [16]. The use of inter-simple sequence repeats (ISSRs) is a molecular fingerprinting technique based on polymerase chain reaction (PCR) that employs sequence primers to amplify DNA sequences contained between microsatellites in genomes [17]. ISSR generally has greater reproducibility and reliability than randomly amplified polymorphic DNA (RAPD) [18], the cost of the analysis is relatively lower than amplified fragment length polymorphisms (AFLP) [19], and there is no need for other DNA sequence information compared to simple sequence repeats (SSRs) [20]. Therefore, ISSR is a powerful tool for informing genetic conservation and sustainable use strategies and has been widely used in genetic diversity studies of species with conservation concerns [21,22,23,24,25], including some orchid species, such as *Amitostigma hemipilioides* [26], *Calanthe tsoongiana* [27], *Cattleya elongate* [28], *Cymbidium goeringii* [29], *Piperia yadonii* [30] and others.

In the present study, we employed ISSR markers to investigate the genetic composition of five natural *C. japonicum* populations distributed in Anhui, Hubei, Zhejiang and Henan province in China with the following specific objectives: (1) to document the level of genetic diversity within *C. japonicum* populations and roughly estimate genetic diversity at the species level; (2) to identify the degree of genetic differentiation among populations to determine factors that have influenced genetic structure; and (3) to discuss the implications for effective conservation according to the basic genetic information.

## 2. Results

### 2.1. Genetic Diversity and Genetic Differentiation

The fourteen selected ISSR primers chosen for analysis produced a total of 109 bands among 128 individuals from five populations. Of these, 42 loci (38.53%) were polymorphic (Table 1). At the population level, the percentage of polymorphic loci (*PPL*) ranged from 7.34% (TMS) to 15.60% (DMS), with an average of 11.19%. The average effective *number of alleles per locus* (*N*_e_) was 1.0728, while the *observed number of alleles* (*N*_a_) was 1.1119. Assuming Hardy–Weinberg equilibrium, *Nei’s gene diversity* (*H*) varied between 0.0297 and 0.0587, with an average of 0.0416, and *Shannon’s information index* (*I*) ranged from 0.0435 to 0.0863, with an average of 0.0613. However, the *H* and *I* values were 0.1273 and 0.1928, respectively, at the species level (Table 1). The species’ *PPL* and *H* were lower than mean level of genetic variation of Orchidaceae (*PPL* = 44.8%, *H* = 0.137) summarized by Hamrick and Godt [31].

According to Nei’s analysis of gene diversity, the percentage of genetic variation among *C. japonicum* populations was 67.12% (*G*_st_) (Table 1), which was substantially higher than the average value of *G*_st_ reviewed in Orchidaceae reviewed by Hamrick and Godt [31], indicating elevated inter-population genetic differentiation and limited gene flow (0.2450). The hierarchical analysis of molecular variance (AMOVA) showed that there was highly significant (*p* = 0.001) genetic diversification among the five *C. japonicum* populations (Table 2). With regard to the total genetic diversity, about 70% was between populations and the rest (30%) resided within populations. The approximate values between *Φ*_pt_ (0.698) from the AMOVA analysis and the *G*_st_ (0.6712) from the POPGENE analysis provide additional support for the statistics used in this study and robustness of the results [16].

**Table 1:** Genetic diversity of *Cypripedium japonicum* populations.

Populations	*N*_a_	*N*_e_	*H*	*I*	*PPL*
**DBS**	1.1284 (0.3361)	1.0670 (0.2128)	0.0393 (0.1194)	0.0593 (0.1738)	12.84%
**SNJ**	1.1101 (0.3144)	1.0724 (0.2282)	0.0413 (0.1252)	0.0609 (0.1810)	11.01%
**TMS**	1.0734 (0.2620)	1.0510 (0.1871)	0.0297 (0.1071)	0.0435 (0.1563)	7.34%
**DMS**	1.1560 (0.3645)	1.1041 (0.2713)	0.0587 (0.1471)	0.0863 (0.2114)	15.60%
**BTM**	1.0917 (0.2900)	1.0697 (0.2274)	0.0389 (0.1248)	0.0563 (0.1794)	9.17%
**Average**	1.1119 (0.3134)	1.0728 (0.2253)	0.0416 (0.1247)	0.0613 (0.1804)	11.19%
**Species Level**	1.3853 (0.4889)	1.2132 (0.3283)	0.1273 (0.1824)	0.1928 (0.2656)	38.53%
	***H*_t_**	***H*_s_**	***G*_st_**	***N*_m_**	
**Mean**	0.1264 (0.0329)	0.0416 (0.066)	0.6712	0.2450	

*N*_a_, observed number of alleles; *N*_e_, effective number of alleles; *H*, Nei’s gene diversity; *I*, Shannon’s information index; *PPL*, the percentage of polymorphic loci; *H*_t_, total genetic diversity; *H*_s_, genetic diversity within populations; *G*_st_, genetic differentiation among populations; *N*_m_, gene flow; values in brackets are standard deviations; DBS, Damingshan population; SNJ, Shennongjia population; TMS, Tianmushan population; DMS, Damingshan population; BTM, Baotianman population.

**Table 2:** Analysis of molecular variance (AMOVA) for the *C. japonicum* populations.

SV	d.f.	SSD	MSD	VC	TVP	*Φ*_pt_	*p* Value
**Among populations**	4	579.734	144.934	5.624	70%	0.698	0.001
**Within populations**	123	299.211	2.433	2.433	30%		
**Total**	127	878.945		8.056	100%		

SV, source of variation; d.f., degree of freedom; SSD, sum of squares; MSD, mean squares; VC, variance component; TVP, total variance percentage; *Φ*_pt_, the proportion of the total variance among populations.

### 2.2. Genetic Relationships and Population Structure

**Figure 1:**
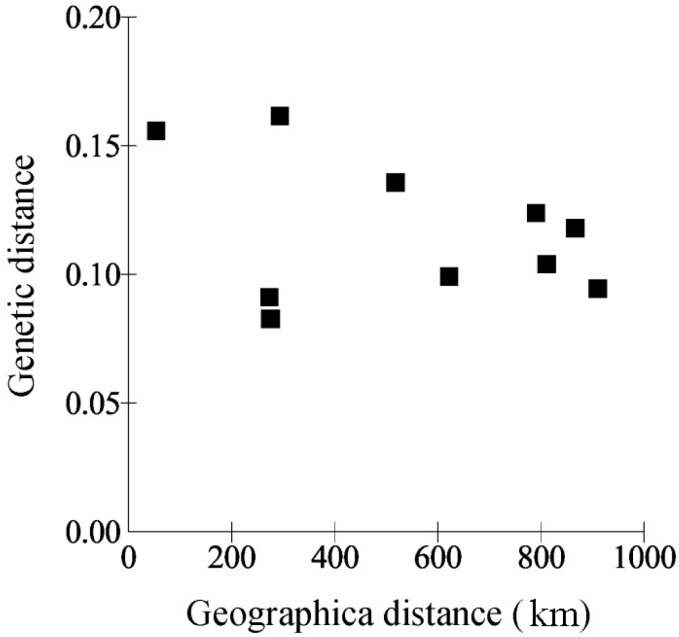
Relationship between genetic and geographic distance of the *C. japonicum* populations.

The genetic distances between *C. japonicum* populations varied from 0.0828 to 0.1616 (Table 3), with an average of 0.1176. The maximum genetic distances and minimum genetic identity were found between the TMS and DBS populations. It is interesting that the second-largest genetic distance (0.1558) was observed in the comparisons of TMS-DMS populations, whose geographical distance is the smallest. These results were consistent with the analysis of isolation by distance with the Mantel test: The genetic and geographic distances did not show any significant correlations in any of the geographic regions (*r* = −0.3429, *p* = 0.8850) (Figure 1).

**Table 3:** Nei’s genetic identity (above diagonal) and genetic distance (below diagonal) among the *C. japonicum* populations.

Populations	DBS	SNJ	TMS	DMS	BTM
**DBS**	****	0.9055	0.8508	0.9128	0.8731
**SNJ**	0.0993	****	0.9098	0.8887	0.9205
**TMS**	0.1616	0.0945	****	0.8557	0.9012
**DMS**	0.0912	0.1180	0.1558	****	0.8835
**BTM**	0.1357	0.0828	0.1040	0.1239	****

****, invalid data.

**Figure 2:**
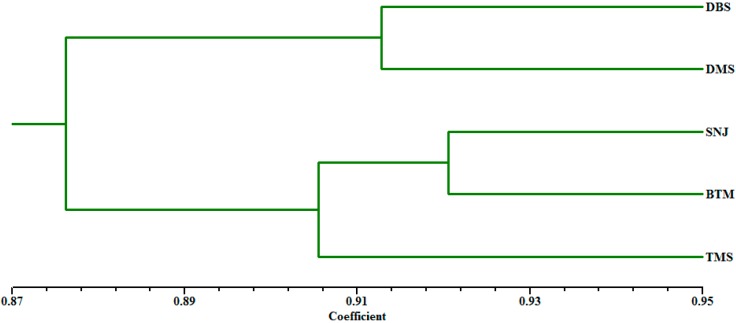
Arithmetic average (UPGMA) dendrogram based on Nei’s genetic distances among populations.

**Figure 3:**
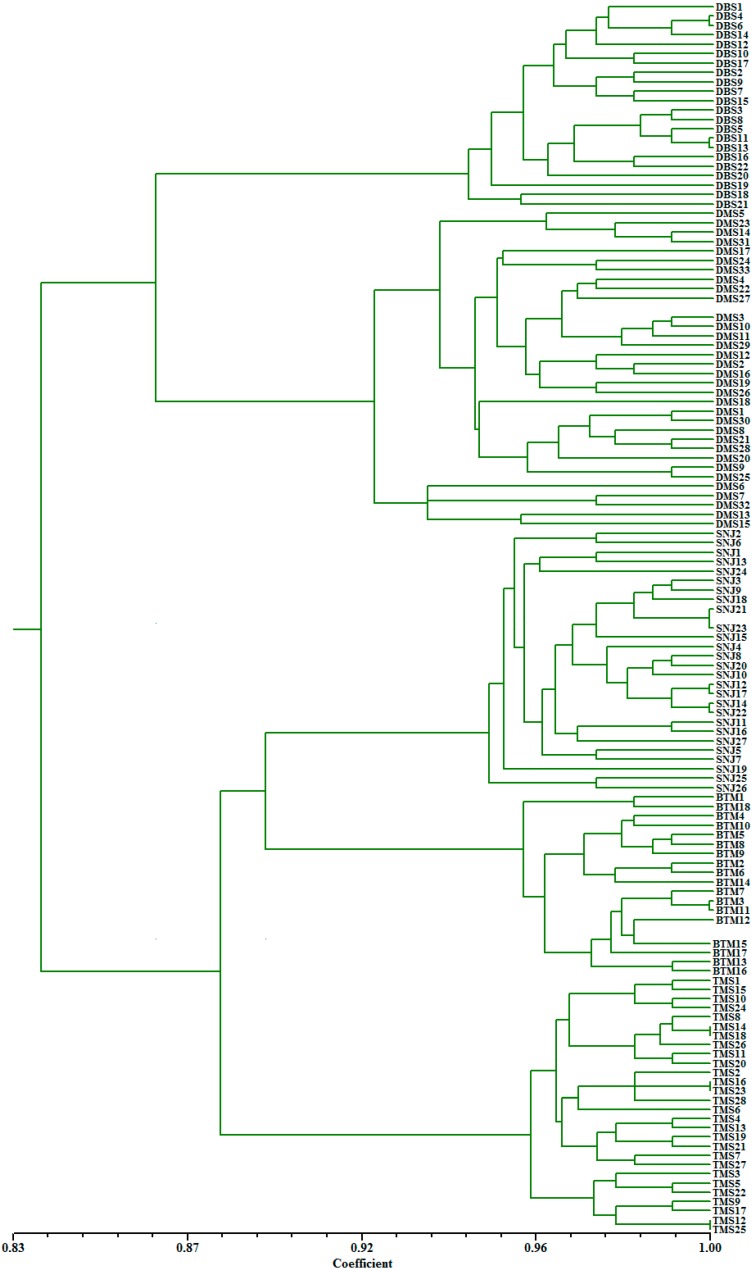
UPGMA dendrogram for all individuals of *C. japonicum*.

The arithmetic average (UPGMA) tree based on Nei’s genetic distance clustered all populations into two major groups (Figure 2). The DBS and DMS populations formed one group, and the remaining three populations of *C. japonicum* formed the other group, which could be divided into two subgroups. One subgroup included the TMS population, and the other contained populations from SNJ to BTM. Furthermore, the dendrogram at the individual level based on Jaccard’s similarity matrix showed that plants from different population origins separated clearly, and individuals within a population were more similar to each other than to those in other populations (Figure 3).

**Figure 4:**
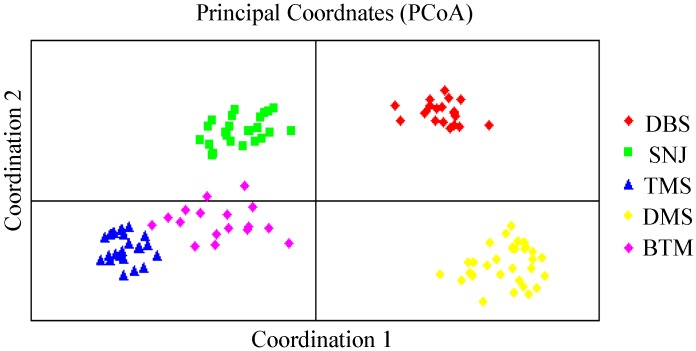
The principal co-ordinate analysis (PCoA) plot of five *C. japonicum* populations based on the two principal axes.

In agreement with the UPGMA cluster analysis, principal coordinate analysis (PCoA) revealed that individuals from each population formed a separate plot and could be clearly distinguished from those of other populations (Figure 4). The individuals formed cohesive clusters in each population. Overall, the populations were grouped in two main groups along Axis 1, which separated individuals of populations SNJ, BTM and TMS from the remaining two populations (DBS and DMS). The first two components accounted for 47.63% (Axis 1 = 30.42%; Axis 2 = 17.21%) of the total variability (Figure 4).

**Figure 5:**
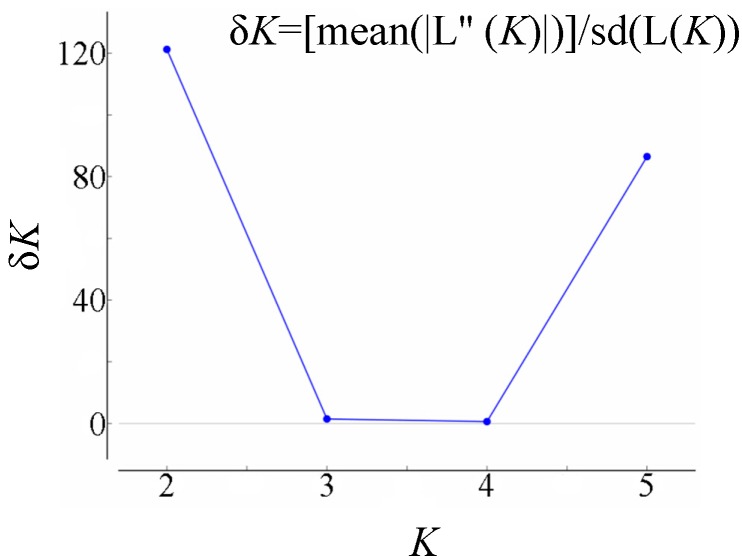
The results of the Bayesian assignment analysis using the Structure Harvester.

**Figure 6:**
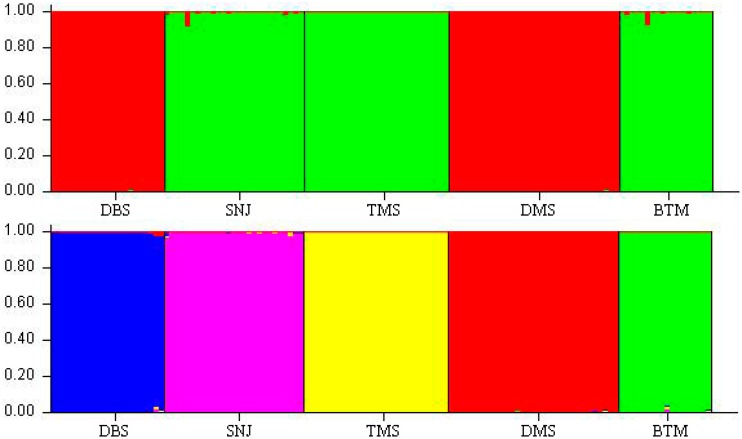
Population structure of six populations of *C. japonicum* prepared using the STRUCTURE program when *K* = 2 (**above**) and *K* = 5 (**below**).

In the ISSR admixture analysis using STRUCTURE, the real *K* value with the highest δ *K* value for the five populations was *K* = 2 (Figure 5), indicating that all of the 128 individuals of *C. japonicum* in the present study shared two genetic pools with few migrants and admixed individuals (Figure 6). The next most likely *K* values (*K* = 5) was shown, as well, indicating that the six populations were isolated relatively independently. The relationships revealed by this program were strongly consistent with that implied by the UPGMA cluster and the PCoA analysis. In addition, we further confirmed the weak association between populations in relation to their geographic location.

## 3. Discussion

### 3.1. Genetic Diversity and Differentiation

*C. japonicum* possessed considerably low genetic diversity both at the species (*PPL* = 38.53%, *H* = 0.1273 and *I* = 0.1928) and population levels (*PPL* = 11.19%, *H* = 0.0416 and *I* = 0.0613) compared to other orchid species (average value: *PPL*_s_ = 64.36%, *H*_s_ = 0.2788 and *I*_s_ = 0.3997; *PPL*_p_ = 50.51%, *H*_p_= 0.1812 and *I*_p_= 0.3084) using the same molecular makers (ISSR) (Table 4). The high *G*_st_ values (0.6712) and the UPGMA and PCoA results suggested limited gene flow, even between the less distant populations (TMS and DMS populations), as well as marked differentiation between populations, which was supported by the AMOVA results (*Φ*_pt_ = 0.698), indicating that the majority of genetic diversity was explained by variation among populations. The species’ ISSR genetic diversity of *C. japonicum* was also lower than some other self-compatible plants (such as *Camellia japonica* [32], *Lilium tsingtauense* [33] and *Viola pubescens* [34]) and asexually reproducing plants (such as *Carex moorcroftii* [35], *Mentha cervina* [16] and *Psammochloa villosa* [36]).

**Table 4:** Comparisons in genetic diversity and differentiation in orchid species based on inter-simple sequence repeat (ISSR) markers.

Orchid Species	*H*_p_	*I*_p_	*PPL*_p_ (%)	*H*_s_	*I*_s_	*PPL*_s_ (%)	*G*_st_ (*F*_st_)	SR
*Amitostigma hemipilioides*	0.603	0.2949	50.9	0.686	0.3873	64.7	0.367	[26]
*Brassavola tuberculata*	-	-	-	0.216	0.314	52.41	-	[37]
*Calanthe tsoongiana*	0.183	0.271	50.0	0.398	0.576	96.80	0.55	[27]
*Cattleya bicolor*	-	-	-	0.219	0.323	56.63	-	[37]
*Cattleya elongata*	-	-	56.8	-	-	-	0.18	[28]
*Cattleya granulosa*	-	-	-	0.163	0.237	30.72	-	[37]
*Cattleya labiata*	-	-	-	0.132	0.193	30.12	-	[37]
*Cattleya schofieldiana*	-	-	-	0.213	0.314	56.02	-	[37]
*Cymbidium goeringii*	0.1945	0.2958	63.06	0.2628	0.4037	88.19	0.244	[29]
*Dendrobium fimbriatum*	0.0871	0.1290	23.93	0.3227	0.4779	89.74	0.7443	[38]
*Gastrodia elata*	0.176	0.270	59.09	0.236	0.367	81.82	0.2725	[39]
*Octomeria crassifolia*	0.2648	0.4005	91.57	0.352	0.530	-	0.76	[40]
*Octomeria grandiflora*	0.2578	0.382	82.4	0.338	0.508	-	0.12	[40]
*Paphiopedilum micranthum*	0.2847	0.4236	80.28	0.3839	0.5646	91.66	0.2577	[41]
*Piperia yadonii (2006)*	0.062	-	-	-	-	-	0.424	[30]
*Piperia yadonii (2007)*	0.059	-	-	-	-	-	0.394	[30]
*Platanthera aquilonis*	0.084	-	22.61	0.184	-	61.7	0.70	[42]
*Platanthera dilatata*	0.1312	-	35.35	0.182	-	57.5	0.49	[42]
*Platanthera huronensis*	0.119	-	32.64	0.172	-	43.0	0.36	[42]
*Tipularia discolor*	0.0309	-	7.95	-	-	-	0.415	[43]
*Average*	0.1812	0.3084	50.51	0.2788	0.3997	64.36	0.4186	

*H*_p_, Nei’s gene diversity at the population level; *I*_p_, Shannon’s information index at the population level; *PPL*_p_, the percentage of polymorphic loci at the population level; *H*_s_, Nei’s gene diversity at species level; *I*_s_, Shannon’s information index at the species level; *PPL*_s_: the percentage of polymorphic loci at the species level; SR, source references; “-” means the corresponding information is not available.

Much of our knowledge of genetic diversity and population structure in *Cypripedium* orchids has come from allozyme markers. Chung *et al*. [14] summarized the allozyme diversity levels of 10 *Cypripedium* species and concluded that genetic diversity within the *Cypripedium* genera can vary substantially, and those with traits, such as wide distribution and broad habitat preference, harbored much greater genetic diversity than rare species with narrower habitat amplitudes. Therefore, commonness and habitat diversity appear to be appropriate predictors for genetic variation in *Cypripedium*. From an ecological perspective, *C. japonicum* is a habitat specialist that is only found on rich humus soils under old, shady, wet deciduous forests on mountainous hillsides, partly due to the narrow mycorrhizal specificity for seed germination and seedling development [44] under local microsite conditions with special soil characteristics.

The biological properties of species, such as the types of reproduction and breeding systems, are stressed as one of the most important factors shaping the genetic diversity and extent of plant population differentiation [8]. Rewarding animal-pollinated species favors near-neighbor mating, which restricts pollen dispersal distances and increases genetic differentiation. [29]. Conversely, a deceptive species generally disperses its pollen over long distances, which results in a high outcrossing rate [1]. *C. japonicum* does not produce food rewards (nectar or edible pollen); rather, it has evolved a food-deceptive pollination system, like most other members of the *Cypripedium* genus [13]. *C. japonicum* probably attracts pollinators visiting the flowers through false nectar guides; The pollinator enters the labellum via a front orifice. To escape, the insect should squeezes out of the rear exit hole, forcing it to pass under the receptive stigma and then pick up a new mass of pollen. Suetsugu *et al.* [13] found that *Bombus ardens* and *Bombus diversus* *diversus* were the effective pollinators in the Japanese *C. japonicum* population, whereas two bumblebee species, *Bombus remotus* and *Bombus imitator*, have been shown to be the effective primary pollinators of *C. japonicum* in the SNJ population. However, the remarkable genetic differentiation among populations in studied *C. japonicum* populations does not match what is expected, indicating that some other more important factors shape their current genetic pattern. The same situation was observed in *Changnienia amoena*, another non-rewarding orchid [1]. We hold that pollen transfer among the studied populations would be very difficult due to large geographic distance and a limited pollinator flying range. Jacquemyn *et al*. [44] suggested that nectar less pollination results in a fitness cost, with declining fruit set and seedling recruitment rates when the population size reduces. Sun *et al*. [11] found a very low fruit set (5.2%–7.7%) of *C. japonicum* individuals under natural conditions. One reason for the low genetic diversity in *C. japonicum* populations can also be explained by the less successful fruit production. The mobility of bumblebees and the highly self-compatibility of *C. japonicum* [13] did not exclude the possibility that they could transport pollinia between flowers within the same genet or between closely-related individuals, which could raise the proportion of recessive deleterious alleles.

Most orchid species have experienced acute declines in abundance due to a wide range of human activities that have resulted in habitat fragmentation, and the remaining populations tend to be small and isolated [45]. Izawa *et al*. [46] pointed out that the habitat range and population size of Lady’s slipper have rapidly declined, especially since the 1950s, mainly because of illegal collecting for the purposes of horticultural use and sale. Small populations of *C. japonicum* were also observed in severely fragmented habitats, which makes this species very vulnerable to habitat disturbance and climate change. Population genetics theory predicts that rare orchid species occurring in small, spatially isolated populations will lead to the random fixation of alleles within populations and subsequent low levels of genetic variation; these populations will exhibit a high degree of population genetic divergence, primarily as a result of stochastic events, genetic drift and inbreeding [14]. Higher levels of genetic drift and inbreeding promote genetic erosion at both the population and species levels. Moreover, fragmented populations, owing to the long distances between *C. japonicum* localities, may be exposed to reduced gene flow, which is also considered to be an important factor increasing genetic divergence between populations. The lack of correlation between geographic and genetic distances of the studied *C. japonicum*, revealed by the Mantel test, UPGMA clustering and PCoA, might be also caused by population fragmentation, genetic drift and restricted gene flow.

Generally, gene flow between populations occurs by one of only two methods: (1) pollen movement by pollinators; and (2) seed dispersal by the wind. Considering the former method, we expect that pollen transfer by insects is not likely when there are large geographical distances between different populations. Long-distance gene flow by pollen is not possible given the limited flight ranges of pollinators. Although orchids are commonly known to show wind seed dispersal over a wide range, our field observations showed that the dense forest structure surrounding the *C. japonicum* populations and the low fruit set likely reduced the probability of seeds being dispersed over long distances between populations. Limited seed dispersal distances have also been reported for other *Cypripedium* orchids. For example, Chung *et al*. [14] stated that most *Cypripedium macranthos* seeds fall close to the maternal plants on the basis of the significant fine-scale spatial genetic structure, and Brzosko *et al*. [47] confirmed that *Cypripedium calceolus* seed dispersal occurred mostly at short distances within or around the fruiting ramets. Infrequent long-distance seed dispersal combined with a paucity of suitable habitats suggests that the natural founding of new *C. japonicum* populations is a rare event. However, we cannot completely exclude great distance gene transfer via seeds through intensive tourism, forest management or some other random event. If long-distance seed dispersal occurs, founder effects are also likely to take place when populations are established by a small number of seed sources, resulting in a loss of genetic diversity during colonization [48]. Therefore, we cannot rule out the possibility that the low levels of genetic diversity, at least in some of the studied populations, may be due to founder effects.

Much literature documenting the influence of the Last Glacial Maximum on population genetic structure describe high levels of genetic diversity in previously unglaciated ranges and relatively low genetic variation in formerly glaciated areas. The regions of sampled *C. japonicum* in this study belong to the “Sino-Japanese Floristic Region”, which was not affected by the major quaternary glaciations [49]. Therefore, the deficiency of genetic diversity in this orchid species contradicts what would be expected. The opposite pattern has also been found in two other congeneric orchids: *Cypripedium parviflorum* [50] and *Cypripedium reginae* [51]. Chung *et al*. [14] attributed the lack of genetic variation both within and among conspecific populations of *C. japonicum* in South Korea to their origination from the same genetically depauperate glacial refugium. However, our data are not sufficient to assess the influence of the Last Glacial Maximum on *C. japonicum* genetic diversity. Further studies involving a wider spectrum of populations from the geographic range of this orchid and the use of more genetic markers, including some codominant DNA markers, could help precisely determine how the glaciations shaped the genetic diversity in its distribution.

### 3.2. Conservation Implication

Knowledge about population genetic structure is considered an important element of conservation biology, so answers to these questions could help develop effective conservation management measures. The loss of genetic diversity will decrease adaptability to environmental changes, which reduces evolutionary potential. In addition, *C. japonicum* has been collected by plant sellers, herbalists and orchid hobbyists in recent decades, due to its horticultural and medicinal potential. This species has suffered from habitat loss and anthropogenic threats, including deforestation, building, agriculture and tourism. Many previously recorded populations have disappeared. Consequently, *C. japonicum* is at risk of extinction in China. Our genetic data and field observations suggest that the main explanatory factors for the low levels of genetic diversity and the shaping of the population genetic structure of *C. japonicum* are narrow habitat preference, genetic drift due to a small and isolated population size, restricted gene flow, historical events and human activity. Considering the current genetic diversity and structure of this orchid species, several conservation guidelines are suggested for the studied *C. japonicum* population.

We recommend that all of the studied populations should be incorporated into conservation plans to maintain their total genetic diversity and, thus, prevent a further decline in the effective population size. Because each individual population comprises a unique genetic pool given the high genetic differentiation among the studied *C. japonicum* populations, the disappearance of each population would remove any unique biological characteristics that it may possess, and this could reduce the overall species biodiversity. Protection of habitats of standing populations *in situ* and prohibition of *C. japonicum* by law should be stressed to prevent habitat damage and harvest pressure on wild populations. Because the fruit set of *C. japonicum* in natural habitats is very low, nearby pollinator (bumblebees) populations and other plant species with floral rewards, if any, should also be safeguarded to ensure reproductive success. Furthermore, the method of artificial gene flow via seeds and hand-mediated artificial pollination suggested for *Cypripedium* *macranthos* var. *rebunense* [46] may be also fit for *C. japonicum* to rapidly increase the gene flow and relieve the effects of genetic drift. However, careful attention should be paid when implementing artificial gene flow. Concerning *ex situ* conservation measures, seed asymbiotic germination and tissue cultures would be helpful for the success of further reinforcement or reintroduction. Sampling for *ex situ* conservation should be taken from as many genets as possible in each population considering the current genetic structure of the studied *C. japonicum*.

To design and implement comprehensive and effective conservation actions for this threatened orchid species, more research is needed to investigate life-history traits, demographic dynamics, germination biology, seedling establishment and other population ecology parameters. In addition, as *C. japonicum* spreads by long, creeping rhizomes, we cannot rule out the possibility that vegetative reproduction could also have a pronounced effect on the genetic structure of populations. Therefore, further study of clonal diversity and structure is also necessary in this endangered orchid.

## 4. Materials and Methods

### 4.1. Study Organism

*Cypripedium japonicum* is a perennial herb with a 10–15-cm underground horizontal rhizome (diameter 3–4.5 mm). It has fibrous fleshy roots, with a diameter up to 2.5 mm. Each growing season, the underground rhizome produces a single new ramet or, more often, a cluster of shoots [11]. This hermaphroditic and terrestrial orchid species is easily characterized by its two nearly opposite, flabellate suborbicular leaves, 10–16 cm long, 10–21 cm wide. The large (sepals and petals are 5–6 cm long), nectarless, solitary yellow-green flower opens in April to May. Individual flowers remain open for 2–3 weeks. The flower has a whitish to yellowish-pink and sac-shaped labellum (length 6–7 cm, width 2–3 cm, depth 2–3 cm) with carmine spots. There are two fertile anthers located above an exit from the labellum. Each anther contains a pollinium, which is a mass of sticky pollen that is usually removed as a unit when an effective pollinator passes through the exit [11]. The fruit that matures in August and September is a nearly spindle capsule up to 5 cm long and about 1.4 cm in diameter and contains thousands of dust-like seeds.

### 4.2. Population Sampling

**Figure 7:**
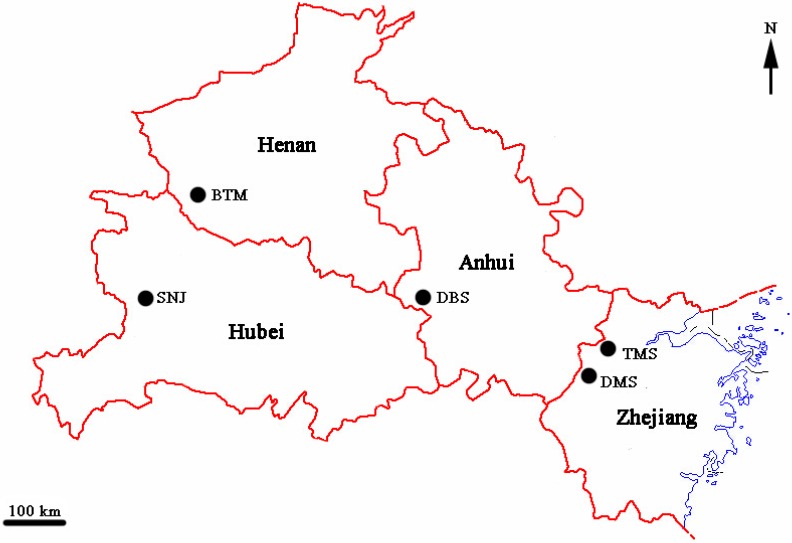
Map of five sampled populations of *C. japonicum* in China.

A total of 128 individuals corresponding to 5 populations with different geographic origins were included in the analysis (Figure 7, Table 5). Samples were collected from one shoot in distinctly spatially isolated patches across each population, taking into account distances between plants and the accompaniment of the plant rhizomes, to avoid harvesting clonal ramets. To minimize damage to this endangered orchid, we only cut 2 cm from the tip of the leaf per plant. Leaf materials were dried with silica gel in zip-lock plastic bags and later stored at −80 °C in the laboratory until DNA extraction. 

**Table 5:** Sampling details of the *C. japonicum* populations.

Populations	Locality	Geographical Coordinate	Sample Size	Voucher Number
DBS	Dabieshan, Anhui province	31°24'N, 116°36'E	22	Q526
SNJ	Shennongjia, Hubei province	31°21'N, 110°03'E	27	Q611
TMS	Tianmushan, Zhejiang province	30°21'N, 119°25'E	28	L605
DMS	Damingshan, Zhejiang province	30°03'N, 118°59'E	33	L522
BTM	Baotianman, Henan province	33°26'N, 111°38'E	18	Q518

### 4.3. Inter-Simple Sequence Repeat (ISSR) Amplification

Total DNA was isolated with Dingguo Plant Genomic DNA Extraction Kits (NEP003-2, Dingguo Biotechnology, Beijing, China) following the manufacturer’s recommendations. The quality and yield of DNA samples were assessed on agarose gels and then precisely measured for DNA concentration using a Quawell Q5000 UV-Vis spectrophotometer (Quawell, San Jose, CA, USA). The DNA samples were diluted to 20 ng/µL in TE buffer (10 mM Tris-HCl (pH 8.0) with 0.1 mM EDTA) and stored at −20 °C until ready for use.

All of the tested primers were synthesized by Sangon Biotech (Shanghai, China). A total of 100 primers (set No. 9, University of British Columbia, Vancouver, BC, Canada) were initially screened, and 14 of them that yielded reproducible and discernible bands were used for the analysis of all 128 samples (Table 6).

**Table 6:** ISSR primers used in the present study.

Primer Name	Sequence (5'→3')	Annealing Temperature (°C)	Primer Name	Sequence (5'→3')	Annealing Temperature (°C)
UBC807	(AG)8T	52	UBC846	(CA)8RT	50
UBC818	(CA)8G	52	UBC849	(GT)8YA	52
UBC827	(AC)8G	53	UBC853	(TC)8RA	51
UBC836	(AG)8YA	51	UBC856	(AC)8YA	50
UBC842	(AG)8G	53	UBC859	(CG)8RC	51
UBC844	(CT)8RC	52	UBC865	(CCG)6	58
UBC845	(CT)8RG	52	UBC868	(GAA)6	47

Y = (C, T); R = (A, G).

PCR reactions were standardized and run in a Vapo-protect Gradient thermal cycler (Bio-Rad, Hercules, CA, USA). Every 25-µL reaction contained 1.5 mM Mg^2+^, 0.5 mM each of dATP, dCTP, dGTP and dTTP, 0.3 µM primer, 30 ng DNA template and 1.5 U Taq DNA polymerase (Aidlab, Beijing, China). A negative control with no DNA template was performed in each reaction to verify the absence of contamination. The amplification conditions were performed with the following program: Initial denaturation at 94 °C for 4 min, 40 cycles of 45 s at 94 °C to denature, 60 s for annealing at the primer-specific melting temperature and 90 s at 72 °C to extend; and the last cycle was followed by a final extension of 10 min at 72 °C. The amplified fragments stained with GelRed (Biotium, Hayward, CA, USA) were separated on 1.8% (*w*/*v*) agarose gels in 1× TBE buffer running at 100 V constant for 1.5 h, and then, the gels were photographed under UV light with a Furi imaging system (FR980, Shanghai, China). Bands with similar migration distances amplified from different individuals were considered homologous. The molecular sizes of the DNA fragments were estimated using a DL2000 ladder (Takara, Shiga, Japan). To ensure the reproducibility of the banding patterns, each PCR amplification and gel running was repeated twice, and only the amplified ISSR products present in both runs were considered.

### 4.4. Statistical Analyses

Each band was assumed to represent the phenotype at a single diallelic locus, because ISSR markers are dominantly inherited [17]. All clearly detectable and consistently reproducible amplified fragments were scored as present (1) or absent (0), and each was treated as an independent character regardless of its intensity. The data obtained were combined in a binary matrix (1/0), and various genetic diversity parameters were calculated using POPGENE version 1.32 [52] under the assumption that the populations were in Hardy–Weinberg equilibrium: *H*, *I*, *N*_a_, *N*_e_, *PPL*, *H*_t_, *H*_s_, *S* and *D*. Population differentiation was analyzed for polymorphisms between populations by *G*_st_, which was estimated by Nei’s gene diversity statistics [53] and Shannon’s information measure [54]. *N*_m_ among these populations was quantified using the formula *N*_m_ = 0.5(1 − *G*_st_)/*G*_st_ [55].

The binary matrix was then employed to perform an individual-level cluster analysis using an UPGMA dendrogram with SAHN (sequential, agglomerative, hierarchical and nested clustering) in NTSYS-pc version 2.20 [56]. Another dendrogram based on Nei’s genetic distance (*D*) was also constructed implementing UPGMA to visualize the genetic relationships among the populations.

To test the correlation between Nei’s genetic distance between populations and geographic distances (in km) among populations, a Mantel test [57] was performed using Tools for Population Genetic Analyses (TFPGA) 1.3 [58] with 999 permutations to determine significance. Geographical distances were calculated by the Google Earth program.

PCoA implemented with the GenAlEx version 6.5 program [59] was carried out to spatially represent the relative genetic distances among individuals and to detect the differentiation consistency between populations defined by the cluster analysis. The hierarchical AMOVA analysis was performed with the same software based on 9999 permutations to obtain the additional measurement of partitioning genetic variation at intra- and inter-population levels.

To identify population structure and genetic admixtures with a Bayesian approach, STRUCTURE v2.3.4 [60] was used to analyze ISSR marker data. This Bayesian method employs individual multilocus genotypes to detect clusters of individuals that minimize Hardy–Weinberg and linkage disequilibria. An admixture model with correlated allele frequencies was performed [61], and default values were maintained for all other parameters. For each value of *K* (from 1 to 5), 10 independent runs were made, and for each run, a Markov chain Monte Carlo (MCMC) of 100,000 iterations was carried out after a burn-in period of 50,000 iterations. Taking the results from the STRUCTURE output file, the most likely value for assigning *K* clusters was determined using the Structure Harvester program [62], which implements the Evanno method [63].

## 5. Conclusions

Our results showed that *C. japonicum* genetic diversity was remarkably low both at the species and population levels and that genetic differentiation between populations was considerably high. The Mantel matrix test revealed no positive correlation between genetic and geographic distances. The UPGMA and STRUCTURE analyses indicated that the five populations can be divided into two main groups. According to genetic data and field observations, narrow habitat preference, genetic drift as a result of a small and isolated population size, limited gene flow, historical factors and human disturbance have shaped the current population structure. Finally, we suggest a combination of conservation strategies, with *in situ* and *ex situ* conservation, to ensure the long-term survival of this endangered orchid species.

## References

[1] Li A., Ge S. (2006). Genetic variation and conservation of *Changnienia amoena*, an endangered orchid endemic to China. Plant Syst. Evol..

[2] Luo Y.B., Jia J.S., Wang C.L. (2003). A general review of the conservation status of chinese orchids. Biodivers. Sci..

[3] Chen S.C., Tsi Z.H. (1998). The Orchid of China.

[4] Trapnell D.W., Hamrick J.L. (2005). Mating patterns and gene flow in the neotropical epiphytic orchid, *Laelia rubescens*. Mol. Ecol..

[5] Sharma I.K., Jones D.L., French C.J. (2003). Unusually high genetic variability revealed through allozymic polymorphism of an endemic and endangered australian orchid, *Pterostylis* aff. *picta* (Orchidaceae). Biochem. Syst. Ecol..

[6] Brzosko E., Wróblewska A., Tałałaj I., Wasilewska E. (2011). Genetic diversity of *Cypripedium Calceolus* in Poland. Plant Syst. Evol..

[7] Wallace L.E. (2002). Examining the effects of fragmentation on genetic variation in *Platanthera leucophaea* (Orchidaceae): Inferences from allozyme and random amplified polymorphic DNA markers. Plant Species Biol..

[8] Brzosko E., Wróblewska A. (2013). Genetic diversity of nectar—Rewarding *Platanthera chlorantha* and nectarless *Cephalanthera rubra*. Bot. J. Linn. Soc..

[9] Swarts N.D., Dixon K.W. (2009). Terrestrial orchid conservation in the age of extinction. Ann. Bot..

[10] Chen S.C., Tsi Z.H., Luo Y.B. (1999). Native Orchids of China in Colour.

[11] Sun H.Q., Cheng J., Zhang F.M., Luo Y.B., Ge S. (2009). Reproductive success of non-rewarding *Cypripedium japonicum* benefits from low spatial dispersion pattern and asynchronous flowering. Ann. Bot..

[12] Subject Database of China Plant http://www.plant.csdb.cn/protectlist.

[13] Suetsugu K., Fukushima S. Pollination biology of the endangered orchid *Cypripedium japonicum* in a fragmented forest of Japan. Plant Spec. Biol..

[14] Chung J.M., Park K.W., Park C.S., Lee S.H., Chung M.G., Chung M.Y. (2009). Contrasting levels of genetic diversity between the historically rare orchid *Cypripedium japonicum* and the historically common orchid *Cypripedium macranthos* in South Korea. Bot. J. Linn. Soc..

[15] Qian X., Li Q.J., Lian J.J., Wang C.X., Tian M. (2013). Genetic diversity of endangered wild *Cypripedium japonicum* populations: An AFLP analysis. Chin. J. Ecol..

[16] Rodrigues L., van den Berg C., Póvoa O., Monteiro A. (2013). Low genetic diversity and significant structuring in the endangered *Mentha cervina* populations and its implications for conservation. Biochem. Syst. Ecol..

[17] Zietkiewicz E., Rafalski A., Labuda D. (1994). Genome fingerprinting by simple sequence repeat (SSR)-floated polymerase chain reaction amplification. Genomics.

[18] Wu C.J., Cheng Z.Q., Huang X.Q., Yin S.-H., Cao K.M., Sun C.-R. (2004). Genetic diversity among and within populations of *Oryza granulata* from yunnan of China revealed by RAPD and ISSR markers: Implications for conservation of the endangered species. Plant Sci..

[19] Yang W., de Oliveira A.C., Godwin I., Schertz K., Bennetzen J.L. (1996). Comparison of DNA marker technologies in characterizing plant genome diversity: Variability in Chinese sorghums. Crop Sci..

[20] Bornet B., Branchard M. (2001). Nonfloated inter simple sequence repeat (ISSR) markers: Reproducible and specific tools for genome fingerprinting. Plant Mol. Biol. Rep..

[21] Farsani T.M., Etemadi N., Sayed-Tabatabaei B.E., Talebi M. (2012). Assessment of genetic diversity of Bermudagrass (*Cynodon dactylon*) using ISSR markers. Int. J. Mol. Sci..

[22] Yang H.Q., An M.Y., Gu Z.J., Tian B. (2012). Genetic diversity and differentiation of *Dendrocalamus membranaceus* (poaceae: Bambusoideae), a declining bamboo species in yunnan, China, as based on inter-simple sequence repeat (ISSR) analysis. Int. J. Mol. Sci..

[23] Golkar P., Arzani A., Rezaei A.M. (2011). Genetic variation in safflower (*Carthamus tinctorious* L.) for seed quality-related traits and inter-simple sequence repeat (ISSR) markers. Int. J. Mol. Sci..

[24] Liu J.F., Shi S.Q., Chang E.M., Yang W.J., Jiang Z.P. (2013). Genetic diversity of the critically endangered *Thuja sutchuenensis* revealed by ISSR markers and the implications for conservation. Int. J. Mol. Sci..

[25] Wei L., Wu X.J. (2012). Genetic variation and population differentiation in a medical herb *Houttuynia cordata* in China revealed by inter-simple sequence repeats (ISSRs). Int. J. Mol. Sci..

[26] Yang Q., Fu Y., Wang Y.Q., Wang Y., Zhang W.H., Li X.Y., Zhang J. (2014). Genetic diversity and differentiation in the critically endangered orchid (*Amitostigma hemipilioides*): Implications for conservation. Plant Syst. Evol..

[27] Qian X., Wang C., Tian M. (2013). Genetic diversity and population differentiation of *Calanthe tsoongiana*, a rare and endemic orchid in China. Int. J. Mol. Sci..

[28] Da Cruz D.T., Selbach-Schnadelbach A., Lambert S.M., Ribeiro P.L., Borba E.L. (2011). Genetic and morphological variability in *Cattleya elongata* Barb. Rodr.(Orchidaceae), endemic to the campo rupestre vegetation in northeastern Brazil. Plant Syst. Evol..

[29] Yao X.H., Gao L., Yang B. (2007). Genetic diversity of wild *Cymbidium goeringii* (Orchidaceae) populations from Hubei based on inter-simple sequence repeats analysis. Front. Biol. China.

[30] George S., Sharma J., Yadon V.L. (2009). Genetic diversity of the endangered and narrow endemic *Piperia yadonii* (Orchidaceae) assessed with ISSR polymorphisms. Am. J. Bot..

[31] Hamrick J.L., Godt M.J.W. (1996). Effects of life history traits on genetic diversity in plant species. Phil. Trans. Roy. Soc. B.

[32] Lin L., Hu Z.Y., Ni S., Li J.Y., Qiu Y.X. (2013). Genetic diversity of *Camellia japonica* (Theaceae), a species endangered to East Asia, detected by inter-simple sequence repeat (ISSR). Biochem. Syst. Ecol..

[33] Guo W., Jeong J., Kim Z., Wang R., Kim E., Kim S. (2011). Genetic diversity of *Lilium tsingtauense* in China and Korea revealed by ISSR markers and morphological characters. Biochem. Syst. Ecol..

[34] Culley T.M., Sbita S.J., Wick A. (2007). Population genetic effects of urban habitat fragmentation in the perennial herb *Viola pubescens* (Violaceae) using ISSR markers. Ann. Bot..

[35] Liu W.S., Wei W., Dong M. (2009). Clonal and genetic diversity of *Carex moorcroftii* on the Qinghai-Tibet plateau. Biochem. Syst. Ecol..

[36] Li A., Ge S. (2001). Genetic variation and clonal diversity of *Psammochloa villosa* (Poaceae) detected by ISSR markers. Ann. Bot..

[37] Fajardo C.G., de Almeida Vieira F., Molina W.F. (2014). Interspecific genetic analysis of orchids in Brazil using molecular markers. Plant Syst. Evol..

[38] Ma J.M., Yin S.H. (2009). Genetic diversity of *Dendrobium fimbriatum* (Orchidaceae), an endangered species, detected by inter-simple sequence repeat (ISSR). Acta Bot. Yunnanica.

[39] Wu H.F., Li Z.Z., Huang H.W. (2006). Genetic differentiation among natural populations of *Gastrodia elata* (Orchidaceae) in Hubei and germplasm assessment of the cultivated populations. Biodivers. Sci..

[40] Barbosa A.R., Silva-Pereira V., Borba E.L. (2013). High genetic variability in self-incompatible myophilous *Octomeria* (Orchidaceae, Pleurothallidinae) species. Braz. J. Bot..

[41] Huang J.L., Li S.Y., Hu H. (2014). ISSR and SRAP markers reveal genetic diversity and population structure of an endangered slipper orchid, *Paphiopedilum micranthum* (Orchidaceae). Plant Divers. Resour..

[42] Wallace L.E. (2004). A comparison of genetic variation and structure in the allopolyploid *Platanthera huronensis* and its diploid progenitors, *Platanthera aquilonis* and *Platanthera dilatata*. Can. J. Bot..

[43] Smith J.L., Hunter K.L., Hunter R.B. (2002). Genetic variation in the terrestrial orchid *Tipularia discolor*. Southeast. Nat..

[44] Shefferson R.P., Weiss M., Kull T., Taylor D.L. (2005). High specificity generally characterizes mycorrhizal association in rare lady’s slipper orchids, genus *Cypripedium*. Mol. Ecol..

[45] Jacquemyn H., Vandepitte K., Brys R., Honnay O., Roldán-Ruiz I. (2007). Fitness variation and genetic diversity in small, remnant populations of the food deceptive orchid *Orchis purpurea*. Biol. Conserv..

[46] Izawa T., Kawahara T., Takahashi H. (2007). Genetic diversity of an endangered plant, *Cypripediummacranthos* var. *rebunense* (Orchidaceae): Background genetic research for future conservation. Conserv. Genet..

[47] Brzosko E., Wróblewska A., Ratkiewicz M., Till-Bottraud I., Nicole F., Baranowska U. (2009). Genetic diversity of *Cypripedium calceolus* at the edge and in the centre of its range in Europe. Ann. Bot. Fenn..

[48] Chung M.Y., Park C.W., Chung M.G. (2007). Extremely low levels of allozyme variation in southern Korean populations of the two rare and endangered lithophytic or epiphytic *Bulbophyllum drymoglossum* and *Sarcanthus scolopendrifolius* (Orchidaceae): Implications for conservation. Biodivers. Conserv..

[49] Qiu Y.X., Fu C.X., Comes H.P. (2011). Plant molecular phylogeography in China and adjacent regions: Tracing the genetic imprints of quaternary climate and environmental change in the world’s most diverse temperate flora. Mol. Phylogenet. Evol..

[50] Wallace L.E., Case M.A. (2000). Contrasting allozyme diversity between northern and southern populations of *Cypripedium parviflorum* (Orchidaceae): Implications for Pleistocene refugia and taxonomic boundaries. Syst. Bot..

[51] Kennedy A.H., Walker G.L. (2007). The population genetic structure of the showy lady’s slipper orchid (*Cypripedium reginae* Walter) in its glaciated and unglaciated ranges. Castanea.

[52] Yeh F., Yang R., Boyle T. (1999). POPGENE version 1.32. Microsoft Window-Based Freeware for Population Genetic Analysis.

[53] Nei M. (1973). Analysis of gene diversity in subdivided populations. Proc. Natl. Acad. Sci. USA.

[54] Lewinton R.C. (1972). The apportionment of human diversity. Evol. Biol..

[55] Nei M. (1987). Molecular Evolutionary Genetics.

[56] Rohlf F.J. (2005). NTSYSpc, Numerical Taxonomy and Multivariate Analysis SystemVersion 2.2 User Guide.

[57] Mantel N. (1967). The detection of disease clustering and a generalized regression approach. Cancer. Res..

[58] Miller M.P. (1997). Tools for Population Genetic Analyses (TFPGA) Version 1.3: A Windows Program for the Analysis of Allozyme and Molecular Population Genetic Data.

[59] Miller M.P., Peakall R., Smouse P.E. (2012). GenAlEx 6.5: Genetic analysis in Excel. Population genetic software for teaching and research—An update. Bioinformatics.

[60] Pritchard J.K., Stephens M., Donnelly P. (2000). Inference of population structure from multilocus genotype data. Genetics.

[61] Falush D., Stephens M., Pritchard J.K. (2003). Inference of population structure using multilocus genotype data: Linked loci and correlated allele frequencies. Genetics.

[62] Earl D.A., von Holdt B.M. (2012). STRUCTURE HARVESTER: A website and program for visualizing STRUCTURE output and implementing the Evanno method. Conserv. Genet. Resour..

[63] Evanno G., Regnaut S., Goudet J. (2005). Detecting the number of clusters of individuals using the software STRUCTURE: A simulation study. Mol. Ecol..

